# *Roseomonas ponticola* sp. nov., a novel bacterium isolated from Pearl River estuary

**DOI:** 10.1099/ijsem.0.004994

**Published:** 2021-10-08

**Authors:** Ling-Zi Yin, Jia-Ling Li, Bao-Zhu Fang, Ze-Tao Liu, Pandeng Wang, Lei Dong, Li Duan, Xiao-Qing Luo, Shan-Hui Li, Wen-Jun Li

**Affiliations:** ^1^​ State Key Laboratory of Biocontrol, Guangdong Provincial Key Laboratory of Plant Resources and Southern Marine Science and Engineering Guangdong Laboratory (Zhuhai), School of Life Sciences, Sun Yat-Sen University, Guangzhou 510275, Guangdong, PR China; ^2^​ State Key Laboratory of Desert and Oasis Ecology, Xinjiang Institute of Ecology and Geography, Chinese Academy of Sciences, Urumqi 830011, PR China

**Keywords:** Pearl River estuary, *Roseomonas ponticola *sp. nov, *Roseomonas*

## Abstract

A novel species of the genus *Roseomonas *, designated SYSU M41301^T^, was isolated from water sample of the Pearl River estuary in Guangdong, China. Polyphasic, taxonomic and phylogenomic analyses were used to determine the taxonomy position of the strain. Phylogenetic analysis using 16S rRNA gene sequence indicated that strain SYSU M41301^T^ showed the highest sequence similarity to *Roseomonas stagni* KCTC 22213^T^ (97.9 %) and *Roseomonas riguiloci* KCTC 23339^T^ (96.4 %). The novel species could be differentiated from other species of the genus *Roseomonas* by its distinct phenotypic and genotypic characteristics. The isolate was Gram-staining-negative, aerobic, short rod-shape, oxidase-positive and non-motile. The predominant respiratory quinone was ubiquinone 8 (Q-8). The major polar lipids were diphosphatidylglycerol, phosphatidylethanolamine, phosphatidylglycerol, phosphatidylcholine, and one unidentified polar lipid. The major fatty acids (>10 % of total) were 11-methyl C_18 : 1_ *ω*7*c*, summed feature 3 (C_16 : 1_ *ω7*c *and*/ or C_16 : 1_ *ω6*c) and summed feature 8 (C_18:  :1_ *ω*7*c* and/or C_18 : 1_ *ω*6*c*). The G+C content of the novel isolate based on genomic DNA was 72.0 mol%. On the basis of phenotypic, genotypic and phylogenetic data, strain SYSU M41301^T^ should be considered to represent a novel species in the genus *Roseomonas,* for which the name *Roseomonas ponticola* sp. nov. is proposed with the type strain SYSU M41301^T^ (=KCTC 72726^T^=CGMCC 1.18613^T^).

The genus *Roseomonas *, which belongs to the family *Acetobacteraceae* of the order *Rhodospirillales* in the *Alphaproteobacteria *, was first proposed by Rihs *et al.* [1]. At the time of writing, the genus *Roseomonas* comprises 48 species and two subspecies with validly published names. Members of this genus are Gram-staining-negative, aerobic and pink pigmented, which have been isolated from various sources. Species of the genus *Roseomonas* were initially isolated from clinical samples that cause infection in children and adults [2]. Members of the genus *Roseomonas* have also been isolated frequently from environment samples, such as drinking water distribution systems [3], freshwater [4], water-cooling system [5], deep-water marine invertebrates [6].

In order to investigate the cultivable planktonic bacterial community in the Pearl River estuary, sea water samples were collected from the Pearl River estuary, Guangdong, China (22° 20′ 56′ N, 113° 44′ 33′ E). The temperature and pH at the sampling site were 28 °C and pH 7.5, respectively. The site also depicted the following chemical parameters during the sampling period (mg l^−1^): NH_4_^+^ (0.1), NO_3_^-^ (0.1), NO_2_^-^ (0.1), TOC (4.7), TON (3.0). During this study, one isolate designated as SYSU M41301^T^ was isolated from estuary sea water sample. The analysis based on 16S rRNA gene sequence of strain SYSU M41301^T^ indicated the isolate belonged to the genus *Roseomonas* with a low sequence similarity identity (<98.0%). This study is an attempt to analyse the phenotypic, chemotaxonomic and molecular characteristics of the novel isolate.

The sample was plated on R2A agar medium and incubated at 28 °C. Distinct colonies were selected and purified on R2A agar medium. One pink-coloured strain SYSU M41301^T^ among the purified isolates showed low 16S rRNA sequence similarity with known members of the genus *Roseomonas *, and was further selected for characterizing of its taxonomic position. Cells of strain SYSU M41301^T^ were maintained in 20 % glycerol (v/v) at –80 °C. The Gram-reaction was tested by the non-staining KOH method [7]. Micromorphology of the cells grown on R_2_A agar for 2 days was observed by using a transmission electron microscope (JEM1400FLASH, JEOL, JAPAN). Cell motility was checked by inoculating the strain in a tube containing semi-solid medium [8]. Growth was also tested on Luria-Bertani (LB) agar, Tryptic Soy Agar (TSA, Difco), nutrient agar and R_2_A agar, following 5 days of incubation at 28 °C. Oxidase and catalase activities were determined by assessing the oxidation of 1.0 % (w/v) tetramethyl-*p*-phenylenediamine [9] and the formation of bubbles upon addition of 3.0 % (v/v) H_2_O_2_, respectively. Growth temperature (4, 14, 23, 28, 37, 45, 50, 55 and 60 °C) and NaCl tolerance (0–15.0 %, at intervals of 0.5 %, w/v) were checked using R_2_A agar for 1 week. The pH range for growth was tested from pH 4.0 to 10.0 (at intervals of 1.0 pH unit, prepared using the buffer system indicated in Nie *et al.* [10]) in R_2_A broth for 1 week. Cellulose, gelatin and starch hydrolysis, H_2_S production, milk coagulation and peptonization, nitrate reduction, Tweens 20, 40, 60 and 80 degradation and urease activity were tested as previously described [11, 12]. Utilization of sole carbon and nitrogen sources were determined as previously described [13]. Other phenotypic characteristics were determined by the API ZYM (bioMérieux), API 20NE (bioMérieux) and GEN III MicroPlate (Biolog) systems using the instructions provided by the manufacturers.

Cells of strain SYSU M41301^T^ were Gram-staining-negative, short rod-shape, and non-motile (Fig. S1, available in the online version of this article). The strain grew well on nutrient agar, R_2_A agar, but not on Luria-Bertani agar and TSA. Colonies on R_2_A agar after 2 days of growth at 28 °C were non-translucent, pink-coloured, circular, entire margin, low convex and smooth, measuring up to 4 mm in diameter. Strain SYSU M41301^T^ was catalase-positive and oxidase-positive. Growth of strain SYSU M41301^T^ was observed at 16–45 °C (optimum, 28 °C), pH 6.0–8.0 (optimum, pH 7.0) and in the presence of 0–5.0 % (w/v) NaCl (optimum 0.5%). The phenotypic characteristics determined by the API ZYM (bioMérieux), API 20NE (bioMérieux) and GEN III MicroPlate system (Biolog) are provided in the Table S1. Detailed phenotypic characteristics were listed in species description, while the comparative analyses with the nearest similarity related type strains in the genus *Roseomonas* were listed in Table 1.

**Table 1.** Differentiating characteristics of strain SYSU M41301^T^ and its closely related type strains of the genus *Roseomonas
* 1. SYSU M41301^T^; 2. *Roseomonas stagni* KCTC 22213^T^; 3 *Roseomonas riguiloci* KCTC 23339^T^ symbols: +, positive; −, negative.

Characteristics	1	2	3
Isolation source	Sea water	Pond water	Fresh water
Cell shape	short-rod	coccobacilli	coccobacilli
Cell size (µm)	0.5×1.0	0.5×1.0	0.5×1.2
Temperature range for growth (°C)	16–45	16–37	16–37
Optimum temperature for growth (°C)	28	28	28
Growth pH	6.0–8.0	4.0–8.0	4.0–8.0
**Utilization of**			
Fructose	−	−	+
Glucose	−	+	−
Glucuronic acid	+	−	−
Lactic acid	−	+	−
Mannose	−	+	−
Sucrose	−	−	+
**API 20NE**			
Aesculin hydrolysis	+	−	−
Nitrate reduction	+	−	−
Urease production	+	−	+
DNA G+C content (mol%)	72.0	72.0	68.0
Fatty acids (>10 %)	11-methyl C_18 : 1_ *ω*7c, summed feature 3, summed feature 8	summed feature 3, summed feature 8	summed feature 3, summed feature 8
Polar lipids	DPG, PG, PE, PC, Ls	DPG, PG, PE, PC, Ls	DPG, PG, PE, PC, Ls

Genomic DNA of strain SYSU M41301^T^ was extracted as previously described [14]. Amplification of the 16S rRNA gene, purification, cloning, sequencing and assembly of the raw sequences were done as described earlier [15]. The sequences obtained were compared with the 16S rRNA gene sequences of species with validly published names in EzBioCloud server database [16]. Sequences of related species in the genus *Roseomonas* were retrieved from the EzBioCloud server database, and multiple alignments performed by using Clustal W in mega version 7.0 software package [17]. Phylogenetic trees were generated with maximum-likelihood [18], neighbour-joining [19] and maximum-parsimony [20] methods by using mega version 7.0 software package [21]. Distance matrix for the neighbour-joining method was generated according to Kimura two-parameter model. Stability of the phylogenetic trees were evaluated by bootstrap analysis [22]. *Elioraea tepidiphila* TU-7T^T^ was used as outgroup.

Whole genome sequencing of strain SYSU M41301^T^ was performed using a paired-end sequencing method with HiSeq X platform (Illumina, San Diego, CA, USA) at Genewiz Company (Guangzhou, China). Reads of each data sets were filtered, and high quality paired-end reads were assembled using SPADES [23]. The COG, KEGG databases were used to annotate the genome sequence. Average nucleotide identity (ANI) values were calculated using JSpecies [24]. The G+C content of the genomic DNA was determined from the genome sequence. Phylogenomic tree was constructed as described by Salam *et al.* [25]. Marker genes were extracted from 21 genomes of strains SYSU M41301^T^, some type species available for genus *Roseomonas* and one outgroup using AMPHORA2 [26]. A total of 31 conserved marker genes *(dna*G, *frr*, *inf*C*, nus*A*, pgk, pyr*G*, rpl*A*, rpl*B*, rpl*C*, rpl*D*, rpl*E*, rpl*F*, rpl*K*, rpl*L*, rpl*M*, rpl*N*, rpl*P*, rpl*S*, rpl*T*, rpm*A*, rpo*B*, rps*B*, rps*C*, rps*E*, rps*I*, rps*J*, rps*K*, rps*M*, rps*S*, smp*B*, tsf*) that are known to present universally were selected. Sequences of each of the marker genes were aligned separately by using muscle [27]. Cleaned alignments were concatenated by using perl script (https://github.com/nylander/catfasta2phyml). Poorly aligned regions were removed from the datasets using Gblocks [28] resulting in a final set of amino acid position that were used in generating the phylogenetic tree with RAxML method [29].

Strain SYSU M41301^T^ showed distant relationships with members of the genus *Roseomonas: Roseomonas stagni* KCTC 22213^T^ (97.9 % sequence identity) and *Roseomonas riguiloci* KCTC 23339^T^ (96.4%). Strain SYSU M41301^T^ formed a distinct clade within the genus *Roseomonas* in the neighbour-joining tree (Fig. 1). This relationship was also supported by the maximum-likelihood (Fig. S2) and maximum-parsimony trees (Fig. S3). Phylogenomic tree (Fig. 2) based on the concatenated alignment of 31 marker genes provides further evidence for the distinct lineage of the strains SYSU M41301^T^. The assembled genome of strain SYSU M41301^T^ has been deposited in the GenBank database under the accession numbers JAERQN000000000, and the raw data of the genome has been deposited under the accession number PRJNA690213. Based on the genome information, strain SYSU M41301^T^ had a genome size of 6 732 422 bp, which were retrieved from 29 contigs. The largest length of the contig was 1 574 521 bp, with N_50_ length of 437 051 bp. The G+C content based on genomic DNA was calculated at 72.0 mol%. For strain SYSU M41301^T^, a total of 4596 genes were predicted, which includes 4488 protein-coding genes and 108 RNA genes. A total of 86.5 % of the genes were assigned a putative function while the remaining protein-coding genes were annotated as hypothetical proteins. COGs (Clusters of Orthologous Groups) categories distributions for the genes are presented in the Table S2. The results of anabolic and catabolic metabolism inferring from Table S2 were consistent with the physiological characterization of strain SYSU M41301^T^. The most abundantly represented CDSs (sequence coding for amino acids in protein) in strain SYSU M41301^T^ were amino acid transport and metabolism (COG category E), followed by inorganic ion transport and metabolism (COG category P) and energy production and conversion (COG category C). Strain M41301^T^ showed positive results for ecological functions and utilization of different carbon sources, so the KEGG database was further used to annotate the genome sequence to identify potential functions (Table S3). Based on the results of this analysis, *nar*G, *nar*Z, *nxr*A, *nir*B, *nas*A, *nir*A, *cys*NC, *cys*H, *cys*J were encoded in the genome of SYSU M41301^T^, which suggested the strain played an important role in the aquatic ecosystem and participated in driving the nitrogen cycle and sulphur cycle. In addition, some carbon sources metabolism related genes including *mae*B, *ppd*K, *tpi*A, *pgm*, *pgi*, *glg*A and other genes have also been found, which implied that strain SYSU M41301^T^ might participate the biogeochemical cycle in the Pearl River estuary.

**Fig. 1.**
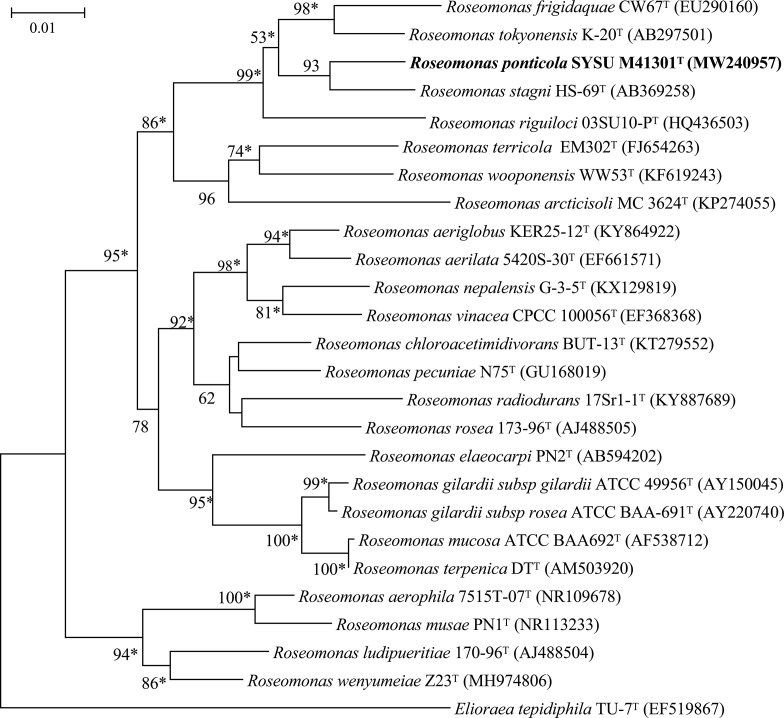
Neighbour-joining phylogenetic tree based on 16S rRNA gene sequences showing the relationship between strain SYSU M41301^T^ and its closest relatives. Bootstrap values (>50%) based on 1000 resamplings are given at the nodes. *Elioraea tepidiphila* DSM 17972^T^ (EF519867) was used as outgroup. Asterisks denote topologies that were also recovered in trees generated with the maximum-likelihood and maximum-parsimony methods. Bar, 0.01 substitutions per nucleotide position.

**Fig. 2.**
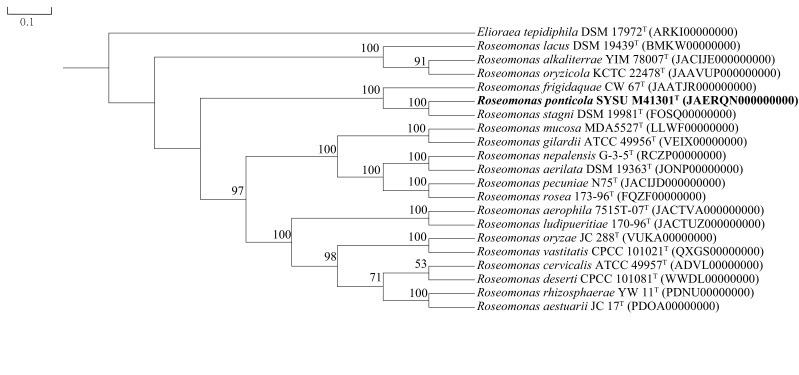
Phylogenetic relationships at genomic level of strain SYSU M41301^T^ and closely related strains of the genus *Roseomonas *. Bootstrap values (>50%) based on 1000 resamplings are given at the nodes. *Elioraea tepidiphila* DSM 17972^T^ (ARKI00000000) was used as outgroup. The relationships were inferred using the RaxML method following concatenation of 31 marker genes present in 21 genomes.

Biomass for study of chemotaxonomic features of strain SYSU M41301^T^ was obtained from cultures grown in R_2_A agar for 7 days (late logarithmic phase of growth). Respiratory quinones were extracted from lyophilized cells [30], and the extract analysed by using a high-performance liquid chromatography (HPLC) [31]. The polar lipids were determined by using a two-dimensional thin-layer chromatography (TLC) procedure on silica gel G 60 plates (Merck) [32, 33]. The staining reagents phosphomolybdate was used for detecting the total lipids, ninhydrin for free-amino group containing lipids, molybdenum blue for phospholipids and α-naphthol for sugar-group containing lipids. Cellular fatty acids were extracted, methylated and identified by using gas chromatography (Agilent Technologies 7890B GC System), following the instructions of the Sherlock Microbial Identification System (MIDI) version 6.1 and the TSBA6 database [34].

The respiratory quinone of strain SYSU M41301^T^ was found to be Q-8. The major polar lipids were identified as diphosphatidylglycerol, phosphatidylethanolamine, phosphatidylglycerol, phosphatidylcholine and one unidentified polar lipid (Fig. S4). The major cellular fatty acids identified were 11-methyl C_18 : 1_ *ω*7*c*, summed feature 3 (C_16 : 1_ *ω7*c and/or C_16 : 1_ *ω6*c) and summed feature 8 (C_18 : 1_ *ω*7*c* and/or C_18 : 1_ *ω*6*c*). A complete fatty acid profile of strain SYSU M41301^T^ is listed in Table S4.

The phylogenetic analysis, morphological and chemotaxonomical characteristics support the characterization of strain SYSU M41301^T^ as a member of the genus *Roseomonas *. The strain can be considered to represent a distinct lineage within the genus *Roseomonas* from its low 16S rRNA gene sequence identities with other members of the genus *Roseomonas* (<98.0 %). In addition, the ANI values between the novel isolate and species in the genus *Roseomonas* were lower than 90.0 %, which are strong evidence for assigning novel isolate into genus *Roseomonas* (Table S5). Besides, strain SYSU M41301^T^ could be differentiated from the nearest species in the genus *Roseomonas* by the features summarized in Table 1. Based on the differentiating characteristics, strain SYSU M41301^T^ represents a novel species of the genus *Roseomonas *, for which the name *Roseomonas ponticola* sp. nov. is proposed.

## Description of *Roseomonas ponticola* sp. nov.

*Roseomonas ponticola* (pon.ti′co.la. L. masc. n. *pontus*, the sea; L. suff. *-cola* (from L. n. *incola*) a dweller, inhabitant; N.L. fem. n. *ponticola*, a dweller of sea)

Cells are Gram-staining-negative, aerobic, non-motile, and short rod-shape, 0.5×1.0 µm. Colonies on Reasoner 2A agar are smooth, circular, opaque and pink in colour after 2 days of cultivation at 28 °C. Growth occurs at 16−45 °C (optimum 28 °C), pH 6.0–8.0 (optimum pH 7.0), and 0–5.0 % (w/v) NaCl (optimum 0.5 %). Positive for milk coagulation and peptonization, hydrolyses urea. Negative for hydrolyses cellulose, gelatin, Tweens 20, 40, 60, and 80 or starch, H_2_S production. Utilizes glucuronamid, *β*-hydroxy-d, l-butyric acid, d-glucuronic acid and tetrazolium blue, but not acetic acid, citric acid, d-fructose, d-fucose, d-galactose, gentiobiose, d-gluconic acid, l-alanine, l-histidine, l-arginine or l-tyrosine. Positive for reduction of nitrates, hydrolyses aesculin, lipase (C14), leucine arylamidase, valine arylamidase, cystine arylamidase, trypsin, *α*-chymotrypsin, *α*-galactosidase. The predominant respiratory quinone is Q-8. The cellular polar lipids are diphosphatidylglycerol, phosphatidylethanolamine, phosphatidylglycerol, phosphatidylcholine, and one unidentified polar lipid. The major fatty acids (>10 % of total) are 11-methyl C_18 : 1_ *ω*7*c*, summed feature 3 (C_16 : 1_ *ω7*c and/or C_16 : 1_ *ω6*c) and summed feature 8 (C_18 : 1_ *ω*7*c* and/or C_18 : 1_ *ω*6*c*). The DNA G+C content of the type strain based on genome is 72.0 mol %.

The type strain SYSU M41301^T^ (=KCTC 72726^T^=CGMCC 1.18613^T^) was isolated from water sample collected from the Pearl River, Guangdong, China. The 16S rRNA gene of strain SYSU M41301^T^ was submitted to GenBank with accession number MW240957. The raw data and assembly genome with accession number PRJNA690213 and JAERQN000000000, respectively.

## Supplementary Data

Supplementary material 1
