# Supplementary material for: Roseomonas ponticola sp. nov., a novel bacterium isolated from Pearl River estuary
Source: Int J Syst Evol Microbiol. 2021 Oct 8;71(10):004994. doi: 10.1099/ijsem.0.004994 (PMC8604164; doi:10.1099/ijsem.0.004994)
Supplement: Supplementary material 1 [file ijsem-71-4994-s001.pdf]

Supplementary materials for publication in IJSEM online

Roseomonas ponticola sp. nov., a novel bacterium isolated from Pearl River estuary

Ling-Zi Yin[1], Jia-Ling Li[1], Bao-Zhu Fang[2], Ze-Tao Liu[1], Pandeng Wang[1], Lei Dong[1], Li Duan[1], Xiao-Qing Luo[1], Shan-Hui Li[1], Wen-Jun Li [1,2*]

[1] State Key Laboratory of Biocontrol, Guangdong Provincial Key Laboratory of Plant Resources and Southern Marine Science and Engineering Guangdong Laboratory (Zhuhai), School of Life Sciences, Sun Yat-Sen University, Guangzhou, 510275, Guangdong, China

[2] State Key Laboratory of Desert and Oasis Ecology, Xinjiang Institute of Ecology and Geography, Chinese Academy of Sciences, Urumqi 830011, PR China

[*]Authors for correspondence:

Wen-Jun Li

Tel. & Fax: +86 20 84111727

E-mail: liwenjun3@mail.sysu.edu.cn

Figure S1. Transmission electron micrograph of SYSU M41301^T after incubation on R_2A for 2 days at 28 ℃.

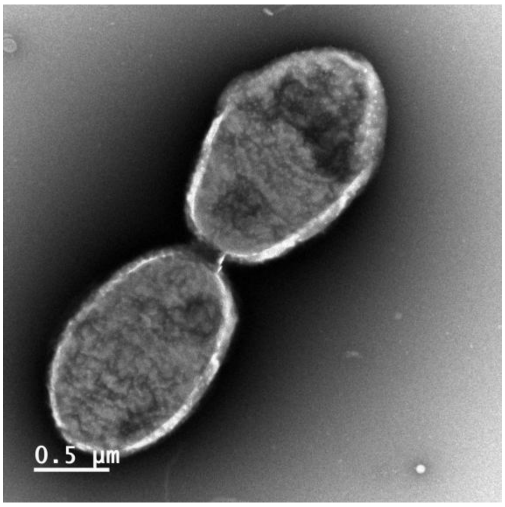

Figure S2. Maximum likelihood tree based on 16S rRNA gene sequences showing the phylogenetic position of strain SYSU M41301^T among the members of the *Roseomonas*. Bar, 0.01 substitutions per nucleotide position. *Elioraea tepidiphila* DSM 17972^T (EF519867) was used as outgroup.

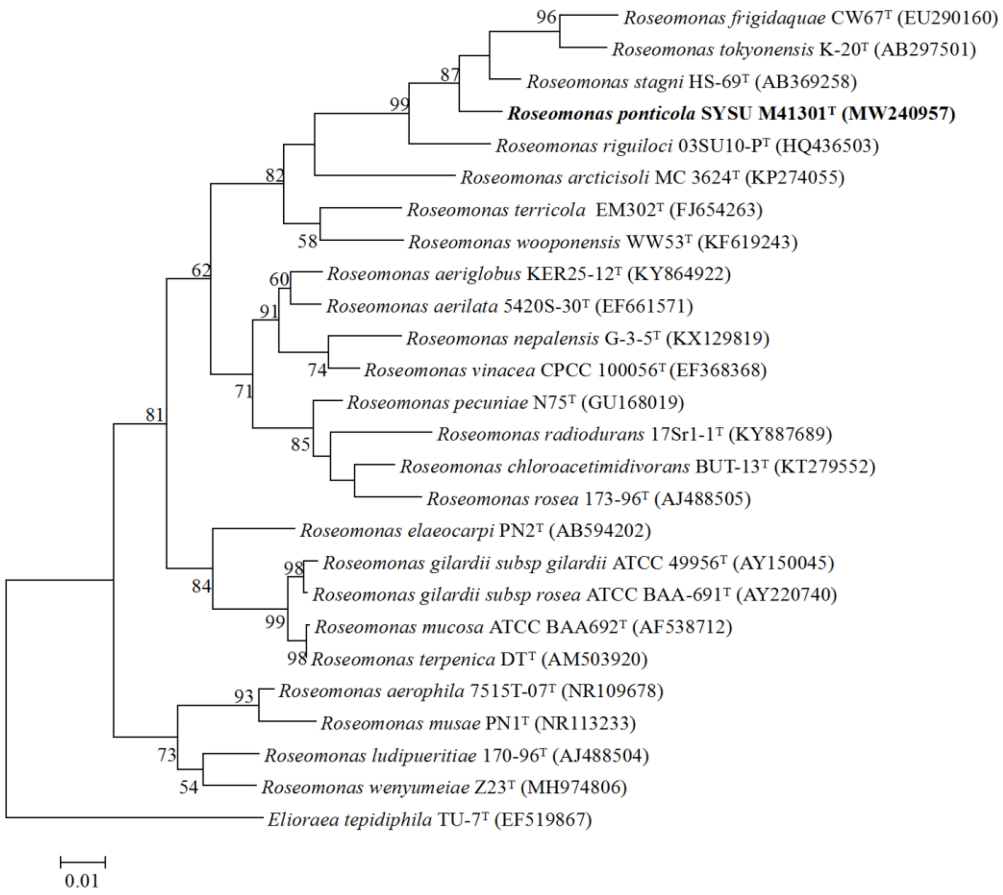

Figure S3. Maximum-parsimony tree based on 16S rRNA gene sequences showing the phylogenetic position of strain SYSU M41301^T among the members of the *Roseomonas*. Bar, 0.01 substitutions per nucleotide position. *Elioraea tepidiphila* DSM 17972^T (EF519867) was used as outgroup.

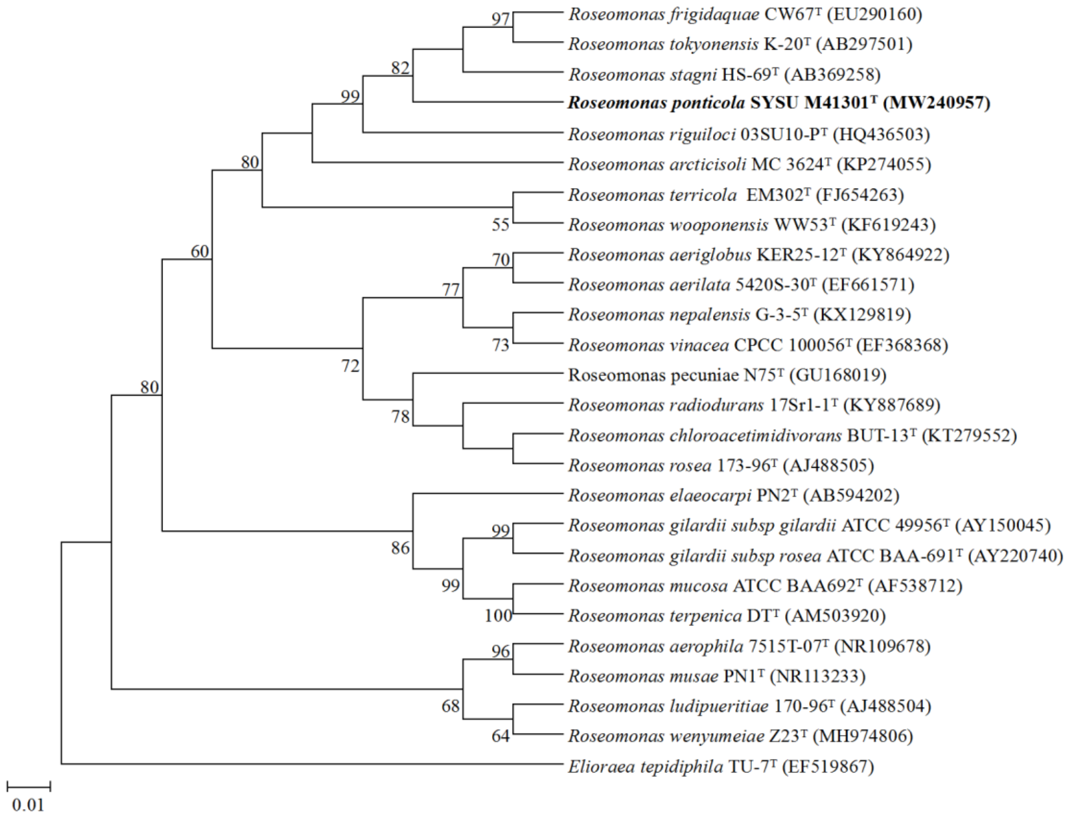

Figure S4. Two-dimensional thin-layer chromatogram of polar lipids of SYSU M41301^{T} and the type strain *Roseomonas riguiloci* KCTC 23339^{T}, *Roseomonas stagni* KCTC 22213^{T}. The chromatographic conditions were as follows: Silica-gel 60 TLC plates (10×10 cm) were spotted with 10.0 µl of a whole-cell polar lipid extract. Chloroform: methanol: water (65: 24: 4, v/v/v) was used in the first direction, and chloroform: acetic acid: methanol: water (80: 18: 12: 5, v/v/v/v) was used in the second direction. Total polar lipids were stained by ethanolic molybdophosphoric acid. Abbreviations: DPG, diphosphatidylglycerol; PE, phosphatidylethanolamine; PG, phosphatidylglycerol; PC, phosphatidylcholine; Ls, unidentified polar lipids.

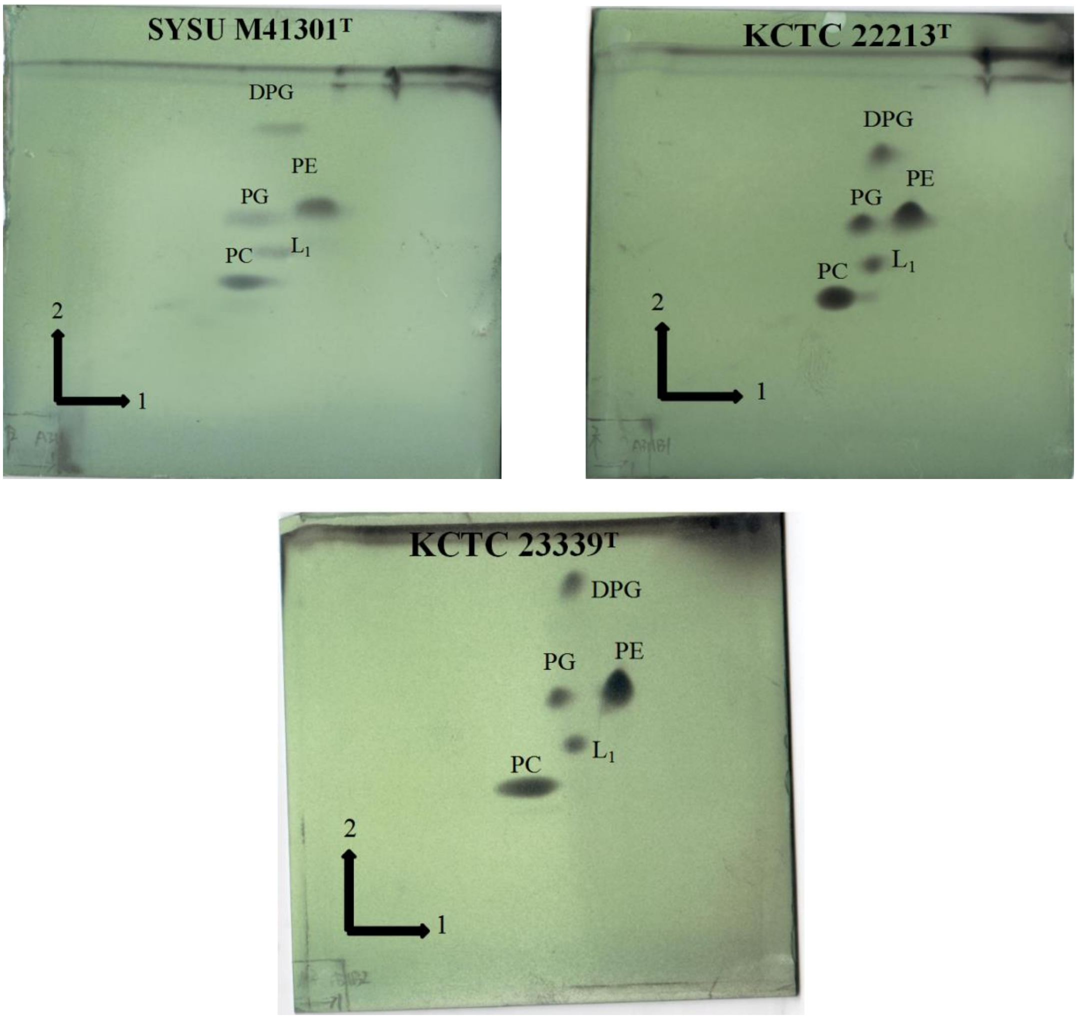

Table S1. Phenotypic characteristics of strain SYSU M41301^{T} determined by Biolog GEN III, API 20NE and API ZYM test kits

BIOLOG GEN III					
Sl. No.	***Characteristic***	***Result***	***Sl. No.***	***Characteristic***	***Result***
	Carbon source utilization assay		36.	L-Pyroglutamic acid	–
1.	Acetic acid	–	37.	*α*-Keto-glutaric acid	–
2.	Acetoacetic acid	–	38.	Glycerol	–
3.	*p*-Hydroxyphenylacetic acid	–	39.	Glycyl-L-proline	–
4.	L-Alanine	–	40.	L-Histidine	–
5.	D-Arabitol	–	41.	Inosine	–
6.	L-Arginine	–	42.	Myo-inositol	–
7.	D-Aspartic acid	–	43.	L-Lactic acid	–
8.	L-Aspartic acid	–	44.	D-Lactic acid methyl ester	–
9.	*γ*-Amino butyric acid	–	45.	*α*-D-Lactose	–
10.	*α*-Hydroxy-butyric acid	–	46.	D-Malic acid	–
11.	*β*-Hydroxy-D,L-butyric acid	–	47.	L-Malic acid	–
12.	*α*-Keto-butyric acid	–	48.	D-Maltose	–
13.	D-Cellobiose	–	49.	D-Mannitol	–
14.	Citric acid	–	50.	D-Mannose	–
15.	Glucuronamid	+	51.	*N*-Acetyl-*β*-D-mannosamine	–
16.	Formic acid	–	52.	D-Melibiose	–
17.	D-Fructose	–	53.	Mucic acid	–
18.	D-Fructose-6-PO_4	–	54.	*N*-Acetyl neuraminic acid	–
19.	D-Fucose	–	55.	Pectin	–
20.	L-Fucose	–	56.	Methyl pyruvate	–
21.	L-Galactonic acid lactone	–	57.	Propionic acid	–
22.	D-Galactose	–	58.	Quinic acid	–
23.	*N*-Acetyl-D-galactosamine	–	59.	D-Raffinose	–
24.	D-Galacturonic acid	–	60.	L-Rhamnose	–
25.	Gelatin	–	61.	D-Saccharic acid	–
26.	Gentiobiose	–	62.	D-Salicin	–
27.	D-Gluconic acid	–	63.	D-Serine	–
28.	*α*-D-Glucose	–	64.	L-Serine	–
29.	3-Methyl glucose	–	65.	D-Sorbitol	–
30.	*N*-Acetyl-D-glucosamine	–	66.	Stachyose	–
31.	D-Glucose-6-PO_4	–	67.	β-Hydroxy-D,LButyric Acid	+
32.	*β*-Methyl-D-glucoside	–	68.	Sucrose	–
33.	Glucuronamide	–	69.	D-Trehalose	–
34.	D-Glucuronic acid	+	70.	D-Turanose	–
35.	L-Glutamic acid	–	71.	Tween 40	–

BIOLOG GEN III (contd.)					
Sl. No.	***Characteristic***	***Result***	***Sl. No.***	***Characteristic***	***Result***
	Chemical sensitivity assay		83.	Niaproof 4	–
72.	pH 5	–	84.	D-Serine	–
73.	pH 6	–	85.	Potassium tellurite	–
74.	1% NaCl	–	86.	Tetrazolium blue	+
75.	4% NaCl	–	87.	Tetrazolium violet	–
76.	8% NaCl	–	88.	Aztreonam	+
77.	Sodium bromate	–	89.	Lincomycin	–
78.	Sodium butyrate	–	90.	Minocycline	–
79.	Lithium chloride	–	91.	Nalidixic acid	–
80.	Guanidine HCl	–	92.	Rifamycin SV	–
81.	Fusidic acid	–	93.	Troleandomycin	–
82.	1% Sodium lactate	–	94.	Vancomycin	–

72

**API 20NE**					
Sl. No.	***Characteristic***	***Result***	***Sl. No.***	***Characteristic***	***Result***
1.	Fermentation of D-glucose	–	***Assimilation of***		
2.	Indole production	–	11.	L-Arabinose	–
3.	Reduction of nitrates to nitrites	+	12.	Capric acid	–
	Reduction of nitrates to N_2	–	13.	Trisodium citrate	–
4.	Arginine dihydrolase	–	14.	Potassium gluconate	–
5.	*β*-Galactosidase	–	15.	*N*-Acetyl glucosamine	–
6.	Hydrolyses aesculin	+	16.	D-Glucose	–
7.	Hydrolyse gelatin	–	17.	Malic acid	–
8.	Urease	+	18.	D-Maltose	–
Assimilation of			19.	D-Mannose	–
9.	Phenylacetic acid	–	20.	D-Mannitol	–
10.	Adipic acid	–			

73

**API ZYM**					
Sl. No.	***Characteristic***	***Result***	***Sl. No.***	***Characteristic***	***Result***
1.	Cystine arylamidase	+	11.	*α*-Glucosidase	-
2.	Leucine arylamidase	+	12.	*β*-Glucosidase	-
3.	Valine arylamidase	+	13.	*β*-Glucuronidase	–
4.	*α*-Chymotrypsin	+	14.	Lipase (C_{14})	+
5.	Esterase (C_4)	-	15.	*α*-Mannosidase	+
6.	Esterase lipase (C_8)	-	16.	Acid phosphatase	-
7.	β-fucosidase	+	17.	β -fucosidase	+
8.	*α*-Galactosidase	+	18.	α -mannosidase	+
9.	*β*-Galactosidase	-	19.	Trypsin	+
10.	*N*-Acetyl-*β*-glucosaminidase	+			

Table S2. Number of genes associated with the general COG functional categories for strain SYSU M41301^{T}

Code	SYSU M41301^{T}		Description
	Value	% age[a]	
C	337	6.3	Energy production and conversion
D	24	0.5	Cell cycle control, cell division, chromosome partitioning
E	753	14.2	Amino acid transport and metabolism
F	94	1.8	Nucleotide transport and metabolism
G	280	5.3	Carbohydrate transport and metabolism
H	165	3.1	Coenzyme transport and metabolism
I	219	4.1	Lipid transport and metabolism
J	202	3.8	Translation, ribosomal structure and biogenesis
K	270	5.1	Transcription
L	148	2.8	Replication, recombination and repair
M	238	4.5	Cell wall/membrane/envelope biogenesis
N	103	4.4	Carbohydrate transport and metabolism
O	133	1.9	Posttranslational modification, protein turnover, chaperones
P	359	3.5	Inorganic ion transport and metabolism
Q	285	6.8	Secondary metabolites biosynthesis, transport and catabolism
R	582	10.9	General function prediction only
S	196	3.7	Function unknown
T	231	4.4	Signal transduction mechanisms
U	54	1.0	Intracellular trafficking, secretion, and vesicular transport
V	44	0.8	Defensive mechanisms
-	610	11.5	Not in COGs

[a]The total is based on the total number of protein-coding genes in the genome

Table S3. Ecological and carbon sources related genes and numbers of strain SYSU M41301^T annotated with KEGG.

Ko id	Ko name	Ko define	SYSU M41301^T
K00370	*nar*G, *nar*Z, *nxr*A	nitrate reductase / nitrite oxidoreductase	1
K00362	*nir*B	nitrite reductase	1
K00372	*nas*A	assimilatory nitrate reductase	1
K00366	*nir*A	ferredoxin-nitrite reductase	1
K02575	NRT, *nar*K, *nrt*P, *nas*A	nitrate/nitrite transporter	1
K00955	*cys*NC	bifunctional enzyme	2
K00390	*cys*H	Phosphoadenosine / phosphosulfate reductase	1
K00380	*cys*J	sulfite reductase (NADPH) flavoprotein	1
K00029	*mae*B	malate dehydrogenase	1
K01006	*ppd*K	pyruvate, orthophosphate dikinase	2
K01803	*tpi*A	triosephosphate isomerase	1
K01835	*pgm*	phosphoglucomutase	1
K01810	*pgi*	glucose-6-phosphate isomerase	1
K00730	*glg*A	starch synthase	1
K01684	*dgo*D	galactonate dehydratase	1
K03381	*cat*A	catechol 1,2-dioxygenase	1
K05549	*ben*A-*xyl*X	benzoate/toluate 1,2-dioxygenase	1

Table S4. Fatty acid profiles of SYSU M41301^{T} and the reference type strains of the genus *Roseomonas*

Strains: 1, SYSU M41301^{T}; 2, *Roseomonas stagni* KCTC 22213^{T};3, *Roseomonas riguiloci* KCTC 23339^{T} All data are from this study.

	1	2	3
**Saturated**			
$C_{12:0}$	1.8	1.1	1.5
$C_{16:0}$	8.8	8.7	9.4
$C_{18:0}$	-	-	2.2
**Branched**			
$C_{18:1}$ 2-OH	3.4	4.9	2.8
**Unsaturated**			
11-methyl $C_{18:1}\omega 7$c	**10.7**	2.3	-
$C_{16:1}\omega 5c$	1.6	1.3	1.5
$C_{18:1}\omega 5$c	1.5	3.6	-
**Summed Feature**			
3	**20.1**	**24.1**	**11.9**
8	**47.5**	**53.1**	**65.4**
9	-	-	1.3

Values are percentages of total fatty acid content. Fatty acids with composition less than 1 % of the total are not listed in the table. Characters in bold letters indicate major fatty acids. Sum Feature 3, $C_{16:1}$ $\omega 7c$ and/or $C_{16:1}$ $\omega 6c$; Sum Feature 8, $C_{18:1}\omega 7c$ and/or $C_{18:1}\omega 6c$; Summed Feature 9, iso-$C_{17:1}\omega 9c$ and/or 10-methyl $C_{16:0}$, Summed Features are fatty acids that cannot be resolved reliably from another fatty acid using the chromatographic conditions chosen. The MIDI system groups these fatty acids together as one feature with a single percentage of the total; -, not detected

Table S5. ANI values of strain SYSU M41301^{T} against the genomes of closest relatives

Note: 1, *Roseomonas aerilata* DSM 19363^{T}; 2, SYSU M41301^{T}; 3, *Roseomonas aerophila* 7515T-07^{T}; 4, *Roseomonas aestuarii* KCTC:22692^{T}; 5, *Roseomonas alkaliterrae* YIM 78007^{T}; 6, *Roseomonas deserti* JCM 31275^{T}; 7, *Roseomonas frigidaquae* KCTC 22211^{T}; 8, *Roseomonas lacus* DSM 19439^{T}; 9, *Roseomonas ludipueritiae* DSM 14915^{T}; 10, *Roseomonas mucosa* NCTC 13291^{T}; 11, *Roseomonas nepalensis* JCM 31470^{T}; 12, *Roseomonas stagni* KCTC 22213^{T}; 13, *Roseomonas rosea* DSM 14916^{T}; 14, *Roseomonas rhizosphaerae* KACC 17225^{T}; 15, *Roseomonas oryzicola* KCTC 22478^{T}

	1	2	3	4	5	6	7	8	9	10	11	12	13	14	15
1	100±0.00	71.75±0.05	71.95±0.15	73.85±0.65	72.60±0.90	72.20±0.10	71.20±0.20	70.80±0.00	72.60±0.20	73.60±0.50	83.35±0.45	71.30±0.00	79.20±0.40	73.65+0.75	71.95±0.25
2	71.75±0.05	100±0.00	72.15±0.05	73.60±0.60	74.85±1.05	72.45±0.15	78.25±0.15	73.00±0.10	72.40±0.30	72.50±0.60	72.60±0.40	88.50±0.00	72.20±0.20	73.70±0.80	74.15±0.25
3	71.95±0.15	72.15±0.05	100±0.00	77.45±0.55	72.55±0.85	76.30±0.10	72.15±0.05	71.45±0.05	79.40±0.00	73.60±0.60	72.75±0.25	71.80±0.10	72.65±0.15	77.45±0.65	72.05±0.15
4	73.85±0.65	73.60±0.60	77.45±0.55	100±0.00	74.70±0.50	79.55±0.65	73.70±0.50	73.00±0.60	77.85±0.45	75.10±0.10	74.70±0.30	73.10±0.60	74.50±0.40	88.30±0.30	73.95±0.35
5	72.60±0.90	74.85±1.05	72.55±0.85	74.70±0.50	100±0.00	73.35±0.85	74.25±0.85	80.00±1.10	72.90±0.70	73.65±0.25	73.45±0.55	74.11±1.01	73.05±0.65	75.05±0.35	82.l0±0.90
6	72.20±0.10	72.45±0.15	76.30±0.10	79.55±0.65	73.35±0.85	100±0.00	72.35±0.05	72.05±0.05	76.30±0.10	74.45±0.55	73.15±0.35	72.05±0.05	73.15±0.15	79.55±0.85	72.90±0.20
7	71.20±0.20	78.25±0.15	72.15±0.05	73.70±0.50	74.25±0.85	72.35±0.05	100±0.00	73.10±0.10	72.30±0.10	72.30±0.50	71.90±0.20	78.00±0.20	71.90±0.10	73.70±0.50	73.65±0.15
8	70.80±0.00	73.00±0.10	71.45±0.05	73.00±0.60	80.00±1.10	72.05±0.05	73.10±0.10	100±0.00	71.60±0.10	71.90±0.60	71.60±0.40	72.70±0.00	71.45±0.15	73.05±0.65	80.50±0.40
9	72.60±0.20	72.40±0.30	79.40±0.00	77.85±0.45	72.90±0.70	76.30±0.10	72.30±0.10	71.60±0.10	100±0.00	74.25±0.45	73.45±0.25	72.20±0.20	73.50±0.00	78.05±0.65	72.30±0.00
10	73.60±0.50	72.50±0.60	73.60±0.60	75.10±0.10	73.65±0.25	74.45±0.55	72.30±0.50	71.90±0.60	74.25±0.45	100±0.00	74.50±0.30	72.10±0.60	74.20±0.40	75.40±0.20	72.85±0.35
11	83.35±0.45	72.60±0.40	72.75±0.25	74.70±0.30	73.45±0.55	73.15±0.35	71.90±0.20	71.60±0.40	73.45±0.25	74.50±0.30	100±0.00	72.10±0.40	79.90±0.00	74.50±0.40	72.85±0.25
12	71.30±0.00	88.50±0.00	71.80±0.10	73.10±0.60	74.11±1.01	72.05±0.05	78.00±0.20	72.70±0.00	72.20±0.20	72.10±0.60	72.10±0.40	100±0.00	71.90±0.20	73.30±0.70	73.15±0.75
13	79.20±0.40	72.20±0.20	72.65±0.15	74.50±0.40	73.05±0.65	73.15±0.15	71.90±0.10	71.45±0.15	73.50±0.00	74.20±0.40	79.90±0.00	71.90±0.20	100±0.00	75.35±0.65	72.40±0.00
14	73.65+0.75	73.70±0.80	77.45±0.65	88.30±0.30	75.05±0.35	79.55±0.85	73.70±0.50	73.05±0.65	78.05±0.65	75.40±0.20	74.50±0.40	73.30±0.70	75.35±0.65	100±0.00	74.00±0.00
15	71.95±0.25	74.15±0.25	72.05±0.15	73.95±0.35	82.l0±0.90	72.90±0.20	73.65±0.15	80.50±0.40	72.30±0.00	72.85±0.35	72.85±0.25	73.15±0.75	72.40±0.00	74.00±0.00	100±0.00
